# Activation of cannabinoid receptor 2 alleviates glucocorticoid-induced osteonecrosis of femoral head with osteogenesis and maintenance of blood supply

**DOI:** 10.1038/s41419-021-04313-3

**Published:** 2021-10-30

**Authors:** Houyi Sun, Weicheng Zhang, Ning Yang, Yi Xue, Tianhao Wang, Hongzhi Wang, Kai Zheng, Yijun Wang, Feng Zhu, Huilin Yang, Wei Xu, Yaozeng Xu, Dechun Geng

**Affiliations:** 1 Department of Orthopedics, The First Affiliated Hospital of Soochow University, Suzhou, 215006 China; 2 Department of Orthopaedics, The First Affiliated Hospital of USTC, Division of Life Sciences and Medicine, University of Science and Technology of China, Hefei, 230000 China; 3 Department of Orthopedics, Changshu Hospital Affiliated to Nanjing University of Traditional Chinese Medicine, Changshu, 215500 China; 4 Department of Orthopedics, The Second Affiliated Hospital of Soochow University, Suzhou, 215006 China

**Keywords:** Prognostic markers, Calcium and phosphate metabolic disorders

## Abstract

In glucocorticoid (GC)-induced osteonecrosis of the femoral head (ONFH), downregulated osteogenic ability and damaged blood supply are two key pathogenic mechanisms. Studies suggested that cannabinoid receptor 2 (CB2) is expressed in bone tissue and it plays a positive role in osteogenesis. However, whether CB2 could enhance bone formation and blood supply in GC-induced ONFH remains unknown. In this study, we focused on the effect of CB2 in GC-induced ONFH and possible mechanisms in vitro and in vivo. By using GC-induced ONFH rat model, rat-bone mesenchymal stem cells (BMSCs) and human umbilical vein endothelial cells (HUVECs) to address the interaction of CB2 in vitro and in vivo, we evaluate the osteogenic and angiogenic effect variation and possible mechanisms. Micro-CT, histological staining, angiography, calcein labeling, Alizarin red staining (ARS), alkaline phosphatase (ALP), tartrate-resistant acid phosphatase (TRAP) staining, TUNEL staining, migration assay, scratch assay, and tube formation were applied in this study. Our results showed that selective activation of CB2 alleviates GC-induced ONFH. The activation of CB2 strengthened the osteogenic activity of BMSCs under the influence of GCs by promotion of GSK-3β/β-catenin signaling pathway. Furthermore, CB2 promoted HUVECs migration and tube-forming capacities. Our findings indicated that CB2 may serve as a rational new treatment strategy against GC-induced ONFH by osteogenesis activation and maintenance of blood supply.

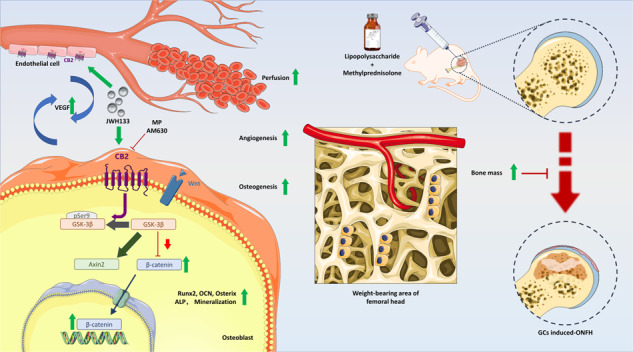

## Introduction

Glucocorticoid (GC)-induced osteonecrosis of the femoral head (ONFH) is a disabling joint disease that could affect patients of all ages, and typically requires total hip arthroplasty as the end-stage treatment [1, 2]. Superphysiological doses of GCs are recognized to be major risk factors of GC-induced ONFH [3, 4]. Over the past decades, GC therapy has been extensively used for spinal trauma, and autoimmune and hematopoietic diseases with its potent anti-inflammatory, immunosuppressive, and metabolic regulator effects [5–7]. Moreover, corticosteroid therapy is now recommended for a large number of patients with severe COVID-19 [8]. However, numerous studies have demonstrated the double-edged sword effect of GCs due to a series of skeletal side-effects including ONFH and osteoporosis [9, 10].

Although the specific pathogenetic mechanism of GC-induced ONFH remains unknown, the pathological process is rather clear. The key manifestation is bone-remodeling disorder in weight-bearing area of femoral head including the destruction and necrotic replacement of normal trabecular bone, resulting in the collapse of the femoral head, which in turn causes progressive degeneration of the entire hip joint [11]. From the perspective of bone metabolism, GC-induced ONFH is a disease of focal bone loss, in which osteogenic activity of osteoblasts play an important role. Studies have found that GC can change the differentiation direction of bone marrow mesenchymal stem cells (BMSCs) by switching from osteogenic differentiation to adipose differentiation [12, 13]. Concurrently, OB maturation and mineralization suppressed by GC intervention [14–16]. There were findings providing evidence that GC-induced bone disease arises from changes in the numbers of bone cells [17]. Thus, teriparatide as osteogenesis booster has been proved to be superior on bone mineral density (BMD) and osteoporotic fracture risk in GC-treated patients [18]. In addition, GCs are also reported to directly damage endothelial cells and impair blood supply to bone tissue [4]. All these factors may ultimately lead to weaken the osteogenesis in GC-induced ONFH.

Cannabinoid receptor 2 (CB2) is a prominent member of the G protein-coupled receptor superfamily [19], and has been demonstrated to be expressed in peripheral nervous system, cardiovascular system, as well as skeletal system [20, 21]. As one of the important research targets in the endocannabinoid system, CB2 regulates immune response and inflammation with various characteristics in different cells and stimulating conditions [22, 23]. Notably, multiple studies have confirmed that CB2 being expressed in osteoblasts and osteoclasts, and is strongly connected to bone-mass regulation. In human studies, CB2 is lower expressed in osteoporotic patients than in healthy bone marrow sample donors [24]. CB2-deficient mice have a significantly accelerated age-related bone loss, CB2 agonist could attenuates ovariectomy-induced bone loss by simultaneous regulation of osteogenesis and osteoclast [25]. For GC-induced bone loss, stimulation of CB2 has been reported to be beneficial for the reducing number and activity of the osteoclasts, as well as bone resorption [26]. CB2 agonists are also reported to be an effective treatment for breast cancer-induced bone loss and pain [27]. Collectively, the effect of CB2 on bone formation has been studied mainly in the field of osteoporosis. CB2 may be a promising target for diagnosis and treatment of bone diseases [28]. Nevertheless, concerning on GC-induced osteogenic inhibition, the role of CB2 remains poorly studied. It is also meaningful to further elucidate whether selective intervention of CB2 had potential protection for GC-induced ONFH.

The regulation of CB2 on osteogenesis may be complex and multidirectional. Researchers observed that CB2-selective agonist partially protected against ovariectomy-induced bone loss by affecting osteoblast differentiation and bone formation through the activation of ERK phosphorylation [29]. Latest study suggested that activation of CB2-induced osteogenic differentiation may involve autophagy induction and p62-mediated Nrf2 deactivation [30]. An interesting recent study found that CB2 promotes kidney fibrosis through orchestrating β-catenin signaling [31]. Given that the Wnt/β-catenin pathway plays a vital role in the regulation of osteogenesis [32], exploring CB2 linked with β-catenin signaling in osteoblasts may be of great value. Equally important is that substantial evidence showed that osteogenesis and focal growth of blood vessels are closely coupled [33], especially in ONFH, which is closely related to blood supply damage. CB2 has been reported to be expressed in vascular endothelial cells, and discussed multiple times in the intervention of tumors and ischemic diseases [34–36]. Therefore, studying vascularization and blood perfusion together with bone formation in femoral head would increase rationality.

This study is focused on the effect of CB2 in GC-induced ONFH and possible mechanisms in vitro and in vivo. We found that low expression of CB2 is associated with superphysiological GC intervention and decreased osteogenesis. By using rat-bone mesenchymal stem cells (BMSCs) and GC-induced ONFH model to address the interaction in vitro and in vivo, we demonstrated that selective activation of CB2 can alleviate GC-induced ONFH with osteogenesis promoted by GSK-3β/β-catenin signaling pathway and blood supply. Our findings may validate a rational new treatment strategy against GC-induced ONFH based on the results of histological, cellular, and molecular level.

## Results

### Activation of CB2 alleviates GC-induced ONFH

**Fig. 1:**
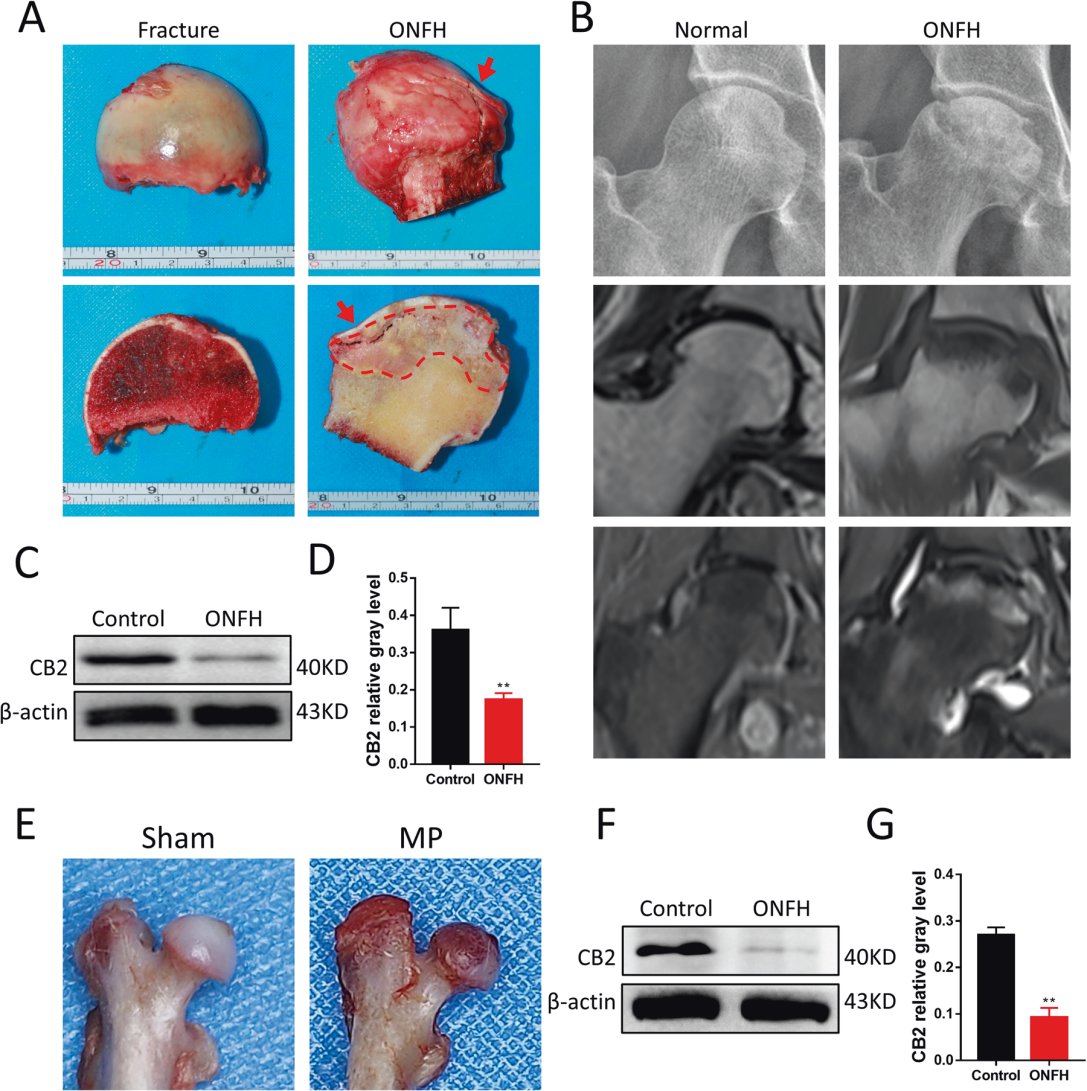
Gross morphology and CB2 expression changes of GC-induced ONFH. **A** Gross morphology of non-ONFH and GC-induced ONFH femoral head. **B** X-ray and MRI images of hip joints. **C**, **D** Expression of CB2 in human femur tissues, *n* = 3 per group. **E** Representative photography of normal and pathological rat femoral heads. **F**, **G** Expression of CB2 in rat femoral head, *n* = 3 per group (*n* = 3 per group, data are shown as mean ± SD, ^**^p < 0.01).

Expression of CB2 is decreased in GC-induced ONFH in femoral-head tissue of both humans and rats. Compared with the control group, the femoral head of steroid-induced femoral-head necrosis showed a significantly abnormal gross appearance (Fig. 1A). For GC-induced ONFH, section plane was consistent with X-ray and MRI images of hip joint (Fig. 1B). Extracting the protein in the femoral-head tissue for western blot suggested that the expression of CB2 in GC-induced ONFH was reduced (Fig. 1C, D). In the rat model established by injection of lipopolysaccharide (LPS) and methylprednisolone (MP), similar gross morphology was also observed (Fig. 1E). In the normal group, the cartilage surface of the femoral head was smooth and uniform in color. The cartilage surface in the weight-bearing area of the model group was abnormal in color and the surface was not smooth. CB2 agonists and inhibitors reduce and aggravate the severity of lesions, respectively. We also kept the organs for hematoxylin and eosin (H&E) staining (Fig. S1).

**Fig. 2:**
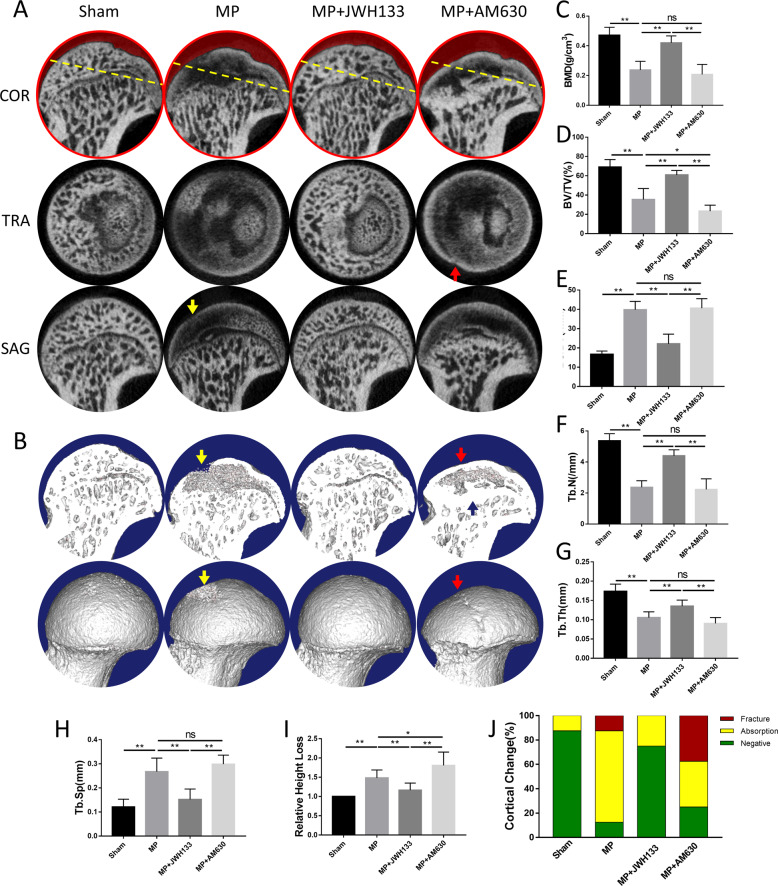
Activation of CB2 attenuate GC-induced bone destruction and maintain femoral-head morphology in GC-induced ONFH rat model. **A** coronal, sagittal, and transverse Micro-CT images. **B** Surface and profile view of femoral heads. Red arrows: cortical fracture; yellow arrows: cortical disruption; blue arrows: compensatory sclerosis. **C** BMD (g/cm^3^). **D** BV/TV (%). **E** BS/BV (1/mm). **F** Tb.N (1/mm). **G** Tb.Th (mm). **H** Tb.Sp (mm), and **I** Relative height loss. **J** Cortical changes (*n* = 10 per group, data are shown as mean ± SD, ^*^p < 0.05; ^**^p < 0.01; ns, not significant).

To clarify the effects of CB2, we established rat model of GC-induced ONFH with, respectively, activation and inhibition of CB2. The results showed that JWH133 ((6aR,10aR)−3-(1,1-Dimethylbutyl)−6a,7,10,10a-tetrahydro-6,6,9-trimethyl-6H-dibenzo[b,d]pyran, selective CB2 agonist) treatment significantly relieved the radiological manifestations of GC-induced ONFH. In order to further explore the morphological changes of the femoral head, we arrange micro-CT scan of the sample, by which cortical and cancellous bone can be visually displayed. From the coronal, sagittal, and cross-sectional view (Fig. 2A), it can be observed that compared with the control group, the model group had severe trabecular bone loss and collapsed in weight-bearing area. JWH133 treatment significantly alleviated the aforementioned femoral-head damage, while the AM630 ((6-Iodo-2-methyl-1-[2-(4-morpholinyl) ethyl]-1H-indol-3-yl)(4-methoxyphenyl)- methanone, selective CB2 antagonist) treatment group also had severe necrosis and collapse. The 3D model of the femoral head reconstructed the bony contours of the femoral heads for each group (Fig. 2B). In the model group and the AM630 treatment group, the absorption and fracture of the cortical bone can be seen, which is consistent with the pathological changes of the human samples. Through the micro-CT analysis of the region of interest (ROI) in the weight-bearing area, we found that the mean BMD of MP group (0.238 ± 0.021 g/cm^3^) was significantly lower than normal group (0.471 ± 0.019 g/cm^3^). By further analysis, we found that JWH133 treatment improved BMD, BV/TV, Tb.N, and Tb.Th compared with the model group, and significantly reduced Tb.Sp and BS/BV (Fig. 2C–H). In addition, JWH133 treatment mitigated the collapse of the femoral head and cortical abnormalities (Fig. 2I, J).

**Fig. 3:**
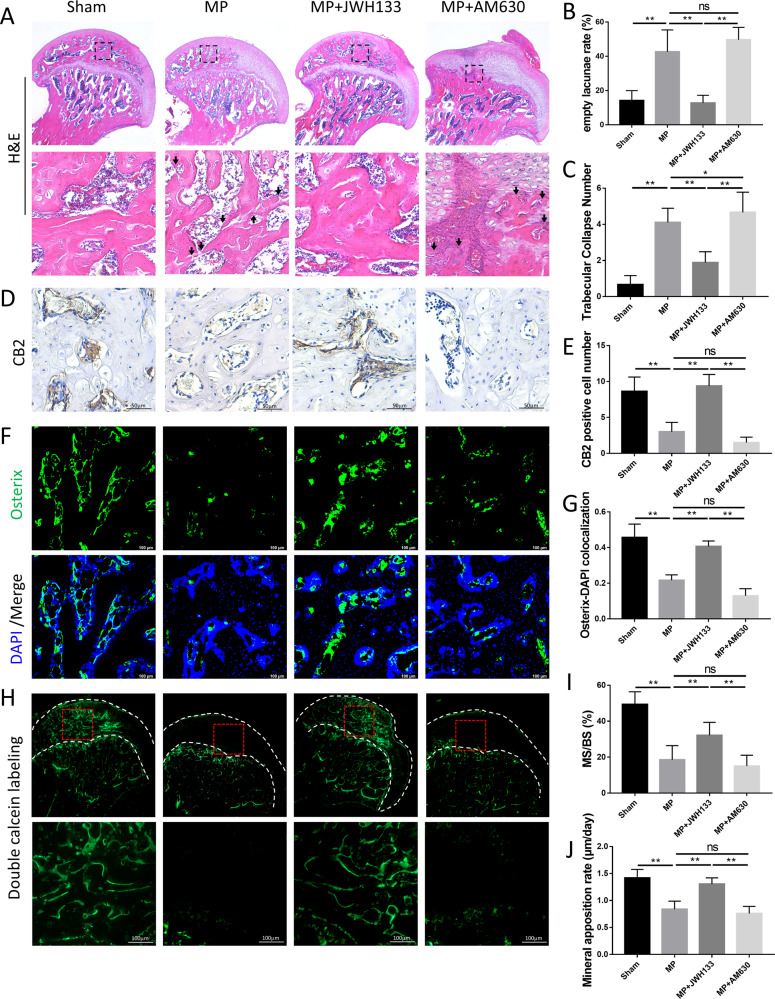
Histological staining, histomorphometric analysis, and CB2 expression in GC-induced ONFH rat. **A**–**C** Hematoxylin and eosin (H&E) staining and histomorphometric analysis. **D**, **E** Immunohistochemical analysis showed significant differences of positive staining. **F**, **G** Immunofluorescence, green (Osterix), and blue (nuclei). **H** Representative images of double calcein labeling. **I** Mineralizing surface/bone surface (%). **J** Mineral apposition rate (μm/day) (*n* = 10 per group, data are shown as mean ± SD, ^*^p < 0.05; ^**^p < 0.01; ns, not significant).

The results of histological staining verify the suppression of bone formation inhibition upon the treatment of JWH133. In H&E staining, higher empty lacunae numbers and more trabecular collapse were observed in MP group, JWH133 significantly changed this trend (Fig. 3A–C). Bone histomorphometry analysis showed that JWH133 greatly maintained bone volume and osteocyte number compared with those in the MP group (Fig. S2A–C), which was consistent with the micro-CT results. Tartrate-resistant acid phosphatase (TRAP) staining showed that the number of osteoclasts increased abnormally in the subchondral bone of the femoral head in the model group, and JWH133 alleviated this phenomenon (Fig. S3A, B). In the TUNEL assay, we observed that osteocytic apoptosis may be significantly promoted by MP, while reduced by JWH133 (Fig. S4A, B). We confirmed the effect of JWH133 and AM630 on CB2 expression by immunohistochemistry (IHC) staining (Fig. 3D, E). JWH133 also significantly reduced the decline of osteogenic indicators Osterix caused by GCs (Fig. 3F, G). The double calcein labeling (Fig. 3H–J) showed that JWH133 treatment increased dynamic bone formation compared with MP group, the mineral apposition rate (MAR) was also enhanced by JWH133. Runx2 and OCN, other important indicators of osteogenic activity levels, also showed similar trends (Fig. S5A–D).

### Effect of CB2 on high-dose GC-induced osteogenesis inhibition in vitro

**Fig. 4:**
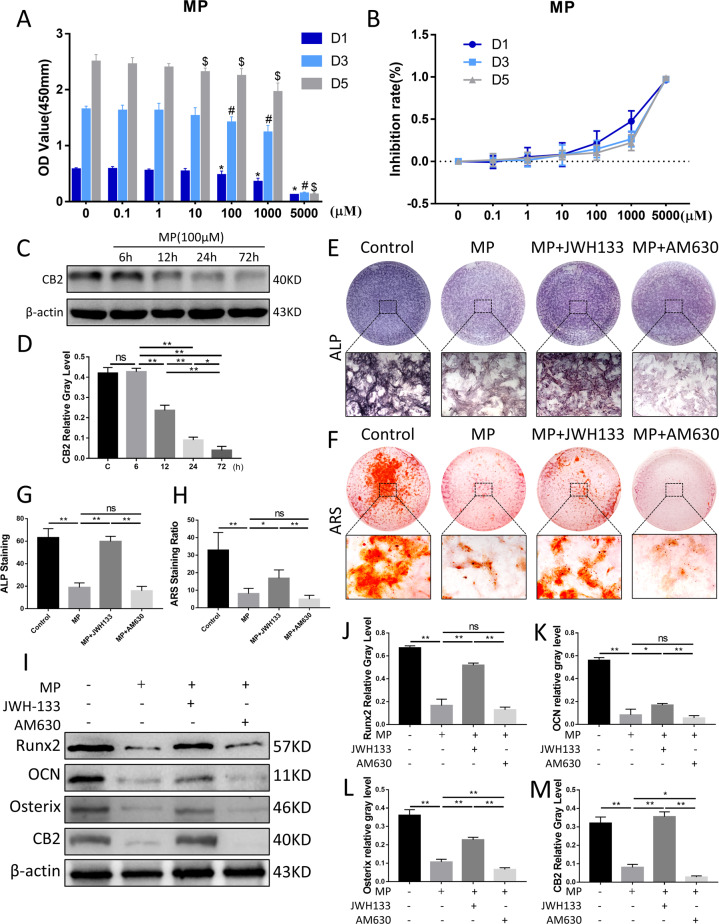
Effects of MP and CB2 treatment on osteogenic differentiation and activity in BMSCs. **A** CCK-8 assay of MP in BMSCs. ^*^p < 0.05, compared with control group (day 1), ^#^*p* < 0.05, compared with control group (day 3), and ^$^*p* < 0.05, compared with control group (day 5). **B** Inhibition rate of BMSCs. **C**, **D** CB2 expression under different duration of MP 100 μM intervention. **E**, **G** ALP staining and analysis. **F**, **H** ARS staining and analysis. **I**–**M** Protein expression levels of Runx2, OCN, Osterix, and CB2 (*n* = 3 per group, data are shown as mean ± SD, ^*^p < 0.05; ^**^p < 0.01; ns, not significant).

To further investigate the effect of CB2 on bone formation under the circumstance of high-dose GC in vitro, we conducted MP, JWH133, and AM630 interventions in BMSCs. CCK8 assay showed that the proliferation and viability of BMSCs was not severely damaged by MP below the concentrations of 100 μM (Fig. 4A, B). With the extension of MP intervention time, the expression of CB2 in BMSCs gradually decreased (Fig. 4C, D). ALP staining showed that CB2 agonists promote the osteogenic activity of BMSCs (Fig. 4E, G), and the alizarin red staining (ARS) results also support the salvage effect of JWH133 treatment on osteogenesis from the perspective of mineralization (Fig. 4F, H). This confirms that MP inhibits osteogenic differentiation regardless of toxicity, and activation of CB2 mitigates this phenomenon. Western blot and quantitative analysis demonstrated that the expression levels of osteogenic markers were significantly increased by JWH133 treatment compared with vehicle group, which indicated that upregulation of CB2 expression alleviated GC-induced osteogenic reduction (Fig. 4I–M). To further explore the effect of AM630 on CB2, we set a combined JWH133/AM630 group compared with JWH133 group. Western blot and quantitative analysis showed that AM630 (1 μM) blunts JWH133 effects on CB2 expression in BMSCs, as well as on osteogenic markers (Fig. S6A–E), suggesting that AM630 could inhibit CB2 signaling in GC-induced ONFH.

### CB2 promotes GC-inhibited osteogenesis via GSK-3β/ β-catenin signaling pathway

**Fig. 5:**
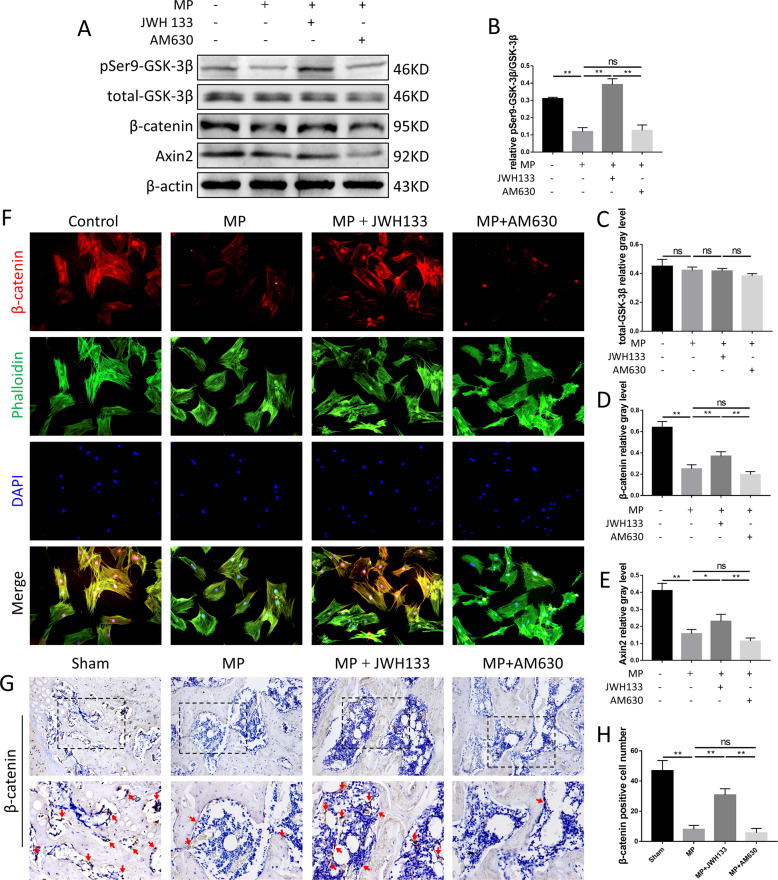
Activity changes of GSK-3β/ β-catenin signaling pathway in vitro and in vivo. **A**–**E** Expression levels of pSer9-GSK-3β, total GSK-3β, Axin2, and β-catenin, *n* = 3 per group. **F** Immunofluorescence staining, red (β-catenin), green (phalloidine), blue (nuclei). **G**, **H** Immunohistochemical staining and analysis for β-catenin, *n* = 10 per group (data are shown as mean ± SD, ^*^p < 0.05; ^**^p < 0.01; ns, not significant).

β-catenin signaling pathway has been established to be a key pathway of osteogenesis. In order to explore the possible mechanism of CB2 in reducing GC-induced osteogenesis inhibition, we evaluated the key markers of related signaling pathways, including GSK-3β/β-catenin signaling pathway. Western blot and quantitative analysis showed that after MP stimulation, the protein expression of pSer9-GSK-3β, Axin2, and β-catenin decreased, while the targeted activation of CB2 significantly reversed the expression of these proteins and pSer9-GSK-3β/GSK-3β (Fig. 5A–D). In the results of cell immunofluorescence staining, similar expression trend was also found. In addition, we observed that the nuclear translocation of β-catenin is promoted by CB2 activation (Fig. 5F). The results of immunohistochemical staining showed that a large number of β-catenin-positive cells exist in normal femoral-head tissue, and GC significantly affected them. The number of positive cells in the JWH133 group was significantly larger than that in the MP group and the AM630 group (Fig. 5G, H).

**Fig. 6:**
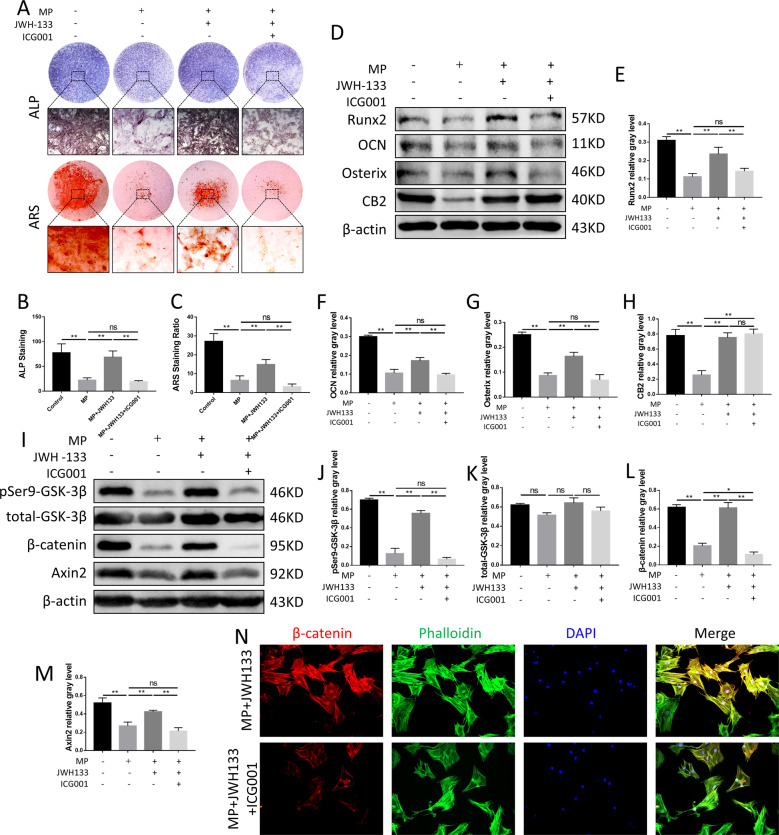
Inhibition of Wnt/β-catenin signaling pathway reversed the osteogenic protective effect of CB2. **A**–**C** ALP, ARS staining and analysis, *n* = 3 per group. **D**–**H** Protein expression levels of Runx2, OCN, Osterix, and CB2, *n* = 3 per group. **I**–**M** Expression levels of pSer9-GSK-3β, total GSK-3β, Axin2, and β-catenin, *n* = 3 per group. **N** Immunofluorescence staining, red (β-catenin), green (phalloidine), blue (nuclei) (data are shown as mean ± SD, ^*^p < 0.05; ^**^p < 0.01; ns, not significant).

To further clarify whether the protection of CB2 on GC-affected osteogenesis by activating the GSK-3β/βcatenin signaling pathway, we used indocyanine green-001 (ICG-001), a specific inhibitor of the Wnt/β-catenin signaling pathway to pretreat cells at a concentration of 10 μM. ALP and ARS staining further verified the inhibitory effect of ICG-001 on JWH133-treated bone formation and mineralization, indicating that ICG-001 restricted the protection of CB2 for bone formation under a high-dose GC condition (Fig. 6A–H).

Western blot and immunofluorescence staining was used to simulate the mechanisms of the phenomenon described above. The results showed that ICG-001 significantly reduced the expression of key markers in GSK-3β/β-catenin signaling pathway (Fig. 6I–M). In addition, the nuclear translocation of β-catenin was also inhibited (Fig. 6N).

### CB2 promotes the maintenance of vascularization and blood perfusion in GC-induced ONFH

**Fig. 7:**
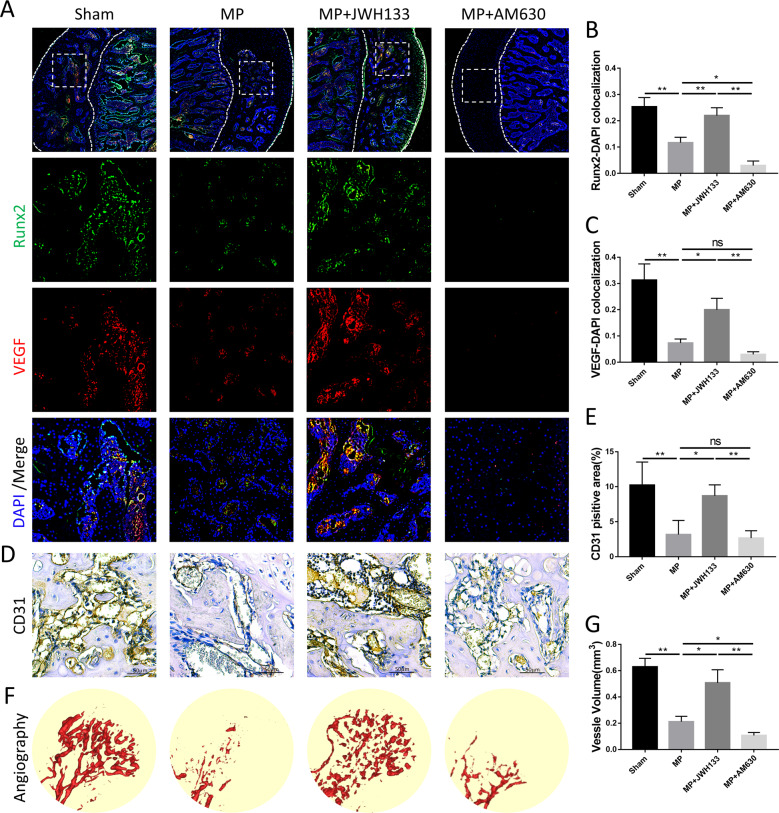
CB2 maintained the vascularization of the femoral head in vivo. **A**–**C** Immunofluorescence, green (Runx2), red (VEGF), and blue (nuclei). **D**, **E** Immunohistochemical staining and quantitative analysis of CD31. **F**, **G** Angiography images of femoral heads analyzed with micro-CT (*n* = 10 per group, data are shown as mean ± SD, ^*^p < 0.05; ^**^p < 0.01; ns, not significant).

Blood supply is also an important aspect of GC-induced ONFH. Interestingly, we found consistency for angiogenesis and osteogenesis in both expression level and histological localization. To explore other possible related factors of CB2 that promote osteogenesis in GC-induced ONFH, we evaluated the levels of femoral-head vascularization under CB2-activating and -inhibiting intervention. Immunofluorescence images of femoral heads showed that the expression level and location of vascular endothelial growth factor (VEGF) is highly consistent with the osteogenic marker Runx2 (Fig. 7A–C). Runx2 and VEGF were both underexpressed in the MP group and the AM630 group, while overexpressed in the control group and JWH133 group. The immunohistochemical staining results showed that the expression of CD31 (Fig. 7D, E) in the model group was significantly lower than that of the control group, and in the JWH133 treatment group it was higher than that in the model group. Vascular imaging is the most convincing method to assess vascular perfusion. We created 3D models of the blood vessels in femoral head and quantified the volume of blood vessels (Fig. 7F, G). The results showed that GC intervention severely damaged the vascular perfusion range and volume of the femoral head, and JWH133 alleviated this phenomenon.

**Fig. 8:**
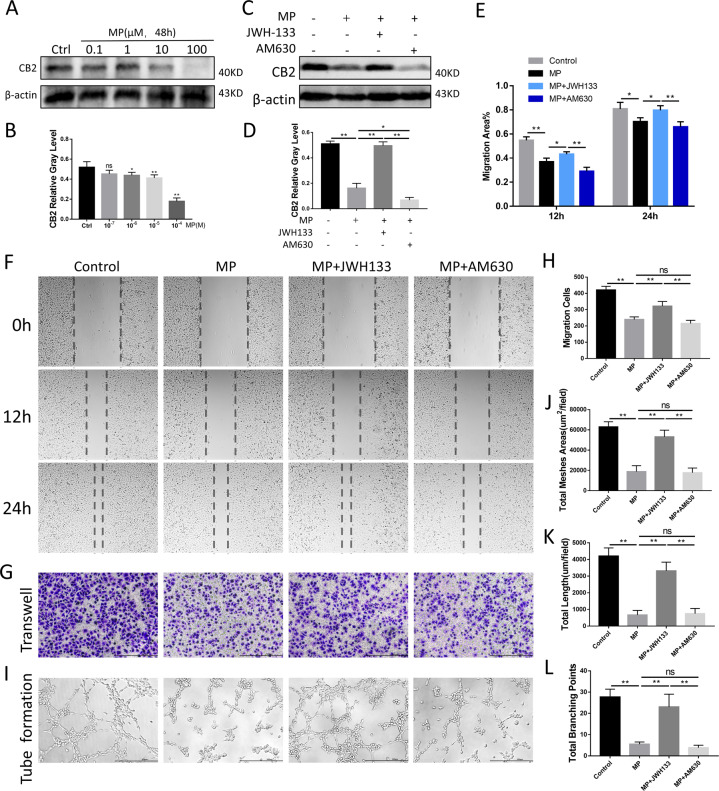
Angiogenesis was promoted by CB2 under MP intervention in vitro. **A**, **B** CB2 expression under different concentration of MP for 48 h. **C**, **D** Expression of CB2 under MP, JWH133, and AM630 intervention. **E**, **F** Scratch assay for 0, 12, and 24 h. **G**, **H** Transwell assay, and migration cells. **I**–**L** Tube formation and quantitative analysis (*n* = 3 per group, data are shown as mean ± SD, ^*^p < 0.05; ^**^p < 0.01; ns, not significant).

To further clarify the effect of CB2 and GCs on angiogenesis, we applied human umbilical vein endothelial cells (HUVECs) to assess endothelial cell function in vitro. We performed CCK8 assay on HUVECs and found that MP concentration was positively correlated with the inhibition rate of HUVECs (Fig. S7A, B). When intervened within safe doses of MP for 48 h, there was also a meaningful CB2 expression difference in HUVECs (Fig. 8A, B). The western blot results showed that CB2 expressions were subjected by MP, JWH133, and AM630 (Fig. 8C, D). The scratch assay clearly showed that at 12 and 24 h, the healing ability of the MP group was significantly slower than that of the normal group, while JWH133 inhibited this effect of MP (Fig. 8E, F). The transwell assay results showed that GC seriously damaged the migration activity of HUVECs, and CB2 agonists relatively made up for this damage (Fig. 8G, H). Tube formation test can directly reflect the ability of blood vessel formation. Compared with the control group, MP showed obvious anti-angiogenesis effect. JWH133 reversed the inhibitory effect of MP on angiogenesis and enhanced the loop-forming ability of HUVECs. The quantitative results displayed the total meshed area, total length, and number of branching points (Fig. 8I–L). The results showed that bevacizumab (0.5 μg/mL) significantly inhibited the migration ability and tube formation activity of HUNECs in JWH133 group, indicating that the effects of CB2 on the HUVEC cells can be blunted by VEGF neutralizing antibody (Fig. S7C-J). These findings indicate that CB2 activation can promote endothelial cell migration and angiogenesis in vitro.

## Discussion

GC-induced ONFH is the ultimate outcome of various factors that affect bone reconstruction. As a disabling disease, it has led to global burden to patients and huge challenges to medical community [37]. A retrospective case investigation based on 6395 ONFH patients in Asian population showed that 24.1% of all ONFHs were caused by GCs, and autoimmune diseases were the main cause of GC-induced ONFH, of which systemic lupus erythematosus (SLE) accounted for the highest proportion [38]. Growing evidence shows that GCs can cause decrease in bone density, and directly or indirectly connected to trabecular abnormalities and replacement by necrotic tissue lacking mechanical strength [18, 39]. The histological appearance of femoral head will not change obviously in the early stage of ONFH, while bone metabolism disorders that may eventually lead to the collapse of the femoral head have already been triggered. Therefore, in symptomatic ONFH, the best opportunity for early-stage intervention may have been missed. Therapeutic GCs themselves actually provided an available therapeutic window that can be seized to prevent the progression of ONFH. So considering the importance of early-stage treatment, studies of GC-induced ONFH seems to carry greater clinical value.

It has been reported that LPS/MP rat model to be an ideal preclinical animal model for GC-induced ONFH [40]. With the help of our modeling program, we have obtained more typical end-stage ONFH pathological manifestations than in previous studies. It proves that the MP combined with LPS modeling method meet the mechanisms of GC-induced ONFH better. CT has unique advantages in the observation of the surface and internal morphology of the femoral head [41]. An inspiring micro-CT study suggested that subchondral fracture begins from the bone resorption area in ONFH [42]. Concentric circles of the femoral head are widely used in clinical research [43], however, it is rarely seen in in vivo studies to evaluate femoral-head collapse with them, which is completely feasible. In the human ONFH, BMD, bone volume, and trabecular thickness were all significantly lower in the collapsed area, which also is the weight-bearing area, on the contrary, in the non-collapsed area, abnormally thickened bone trabeculae were observed [44]. In our micro-CT analysis, ROIs are also set in the subchondral area of the weight-bearing area. Bone loss, compensatory sclerosis, cortical fracture, and resorption were observed in GC-induced ONFH, and CB2 agonist alleviated the exception. The results of histological staining also confirmed this phenomenon. While analyzing the difference in the weight-bearing area, we observed evident bone loss. Unexpectedly, abnormally sclerotic bone area appeared just under the collapsed area, which is consistent with the aforementioned human study.

Previous studies have proved that the existence of endocannabinoid system in bones, blood vessels, spleen, intestines, brain, and many peripheral tissues [45]. Compared with CB1, GPR55, GPR119, TPRV1, TPRV4, and other related receptors, the expression level of CB2 is higher in bone tissue [46]. Selective CB2 agonists have also been shown to play a role in diseases such as colon cancer [47], inflammatory bowel disease [48], and atherosclerosis [49]. Our results confirmed the protective effect of CB2-selective agonist on GC-induced bone loss. The activation of CB2 promotes the osteogenic differentiation of BMSCs, increased ALP activity, and enhanced calcification in the extracellular matrix. The expression of osteogenic markers including Runx2, OCN, and Osterix was promoted by CB2 in vivo and in vitro.

One explanation for this is, CB2 rescued GCs inhibition of GSK-3β/β-catenin signaling pathway. GCs can affect osteogenic differentiation and downregulate the expression of Runx2 and ALP, thereby inhibiting bone formation in vitro and in vivo [50, 51]. The Wnt/β-catenin signaling pathway is one of the mechanisms by which GCs depress bone formation [52]. In our study, GCs decreased the level of β-catenin as well as its target Axin2, however, upregulated CB2 reversed the expression of Axin2 and β-catenin. Also, JWH133 increased pSer9-GSK-3β/GSK-3β, resulting in reduced inactivation and degradation of β-catenin. In addition, BMSCs’ immunofluorescence staining indicated that nuclear translocation of β-catenin is also promoted by CB2 agonists. We reversely verified the results on the β-catenin pathway with the inhibitor ICG-001. The upstream and downstream regulation of this effect was not included in this study. Through literature, upstream factor including microRNA-187-3p may be the osteogenic regulator’s effect of CB2 [53], and β-arr1/Src complex may be involved in the connection between CB2 and GSK-3β/β-catenin signaling pathway [31].

Considering that bone is a highly vascularized tissue, the coupling of osteogenesis and angiogenesis events closely affects bone reconstruction [33, 54]. Studies in the field of oncology have proved GCs’ anti-angiogenic properties by damaging the migration ability of endothelial cells [55]. Hypercoagulable state and abnormal microthrombus formation were induced by GCs in the necrotic region of the femoral head [4]. Thus, we gave plenty of attention to vascularization and blood perfusion in GC-induced ONFH. Many studies focusing on angiogenesis of ONFH put osteogenesis and angiogenesis pathologically juxtaposed [56–58], which does make sense. Our point of view is that from the perspective of disease progression, vascularization and blood perfusion should be considered as the precursor to bone formation, which is also supported by literature [59].

By activating CB2, the ability of HUVECs migration and tube formation was protected from high-dose GCs. In vivo, the expression of VEGF, CD31, and the volume of blood vessels was significantly reduced, suggesting that GCs caused severe devascularization of femoral head, and JWH133 brought a compensation. The colocalization staining of Runx2 and VEGF proved that vascularization and osteogenesis are indeed strongly associated in GC-induced ONFH. We did not arrange any devascularization experiment, because countless avascular necrosis has made this conclusive. Endocannabinoid system may importantly participate the regulation of vascular status in a complicated way [60]. Pharmacologically, outcomes varies in different tissues and diseases [61]. Studies showed hypoxia-inducible factors (HIFs) and VEGF regulate osteogenesis–angiogenesis coupling [62], and VEGF directly reflects angiogenesis and indirectly stimulates osteogenesis [63]. The effect of CB2 on the blood vessels of the femoral head may also be based on VEGF. Wnt/β-catenin signaling also plays a role in the angiogenic activity of endothelial cells, but may be with controversy in femoral head [64–66].

A multiple hit theory exists in GC-induced ONFH, the more risk factors, the higher incidence for ONFH. Inflammation and osteoclast activity may also participate in bone resorption. The role of CB2 agonists in anti-inflammatory and inhibiting osteoclasts has also been reported [67]. Other factors of the neuro-endocrine axis, including CB1, may affect GC-induced ONFH by regulating bone metabolism at multiple levels [68]. Moreover, other meaningful signaling pathways are worth exploring in further study. We hypothesized that the secondary-arthritis changes of acetabular would appear by severely collapsed femoral head, further studies are awaited. In addition, ethical and legal issues need to be carefully considered about use of ECS components.

## Conclusions

CB2 alleviates GC-induced ONFH by attenuating the GC-induced osteogenesis inhibition and blood supply. CB2 plays a role in osteogenic protection from GCs through the GSK-3β/β-catenin signaling pathway. By promoting endothelial cell migration and VEGF level, CB2 stimulated angiogenesis and local blood perfusion. CB2 may be a potential therapeutic target for GC-induced ONFH in the future.

## Materials and methods

### Animals and grouping

Approved by the Animal Ethics Committee of the First Affiliated Hospital of Soochow University, all experimental procedures were completed under the guidance of Care and Use of Laboratory Animals. A total of 60 male Sprague-Dawley (SD) rats (10 weeks old, 300 ± 30 g) were obtained from the Laboratory Animal Center of Soochow University (Soochow, China), and were randomly divided into four groups. Sham group were used as control, GC-induced ONFH models were established in the other three groups: model group, CB2-agonist treatment group (JWH133), and CB2-antagonist treatment group (AM630).

### Model setup and drug treatment

GC-induced ONFH models were constructed as the following steps. The rats were weighed before the drug dose. Daily intraperitoneal (i.p.) injection of LPS (20 μg/kg, Sigma-Aldrich) was given for the first 3 days. Then an intramuscular (i.m.) dose of methylprednisolone (MP, 60 mg/kg, Pfizer) was administered each day for the next 4 days. MP was injected into the left and right gluteus muscles alternately. CB2-agonist group was treated with JWH133 (1 mg/kg/day, Tocris Bioscience) via i.p. injection, CB2-antagonist group was treated with AM630 (1 mg/kg/day, Tocris Bioscience), both groups were administered daily for 3 consecutive weeks from the second day after MP injection. The control group received physiological saline. Animals were sacrificed for femoral heads 4 weeks after MP medication.

### Micro-CT analysis

To assess the imaging changes, the femoral heads of rats were scanned and analyzed using the high-resolution micro-CT SkyScan 1176 (Bruker, Aartselaar, Belgium). Scanning parameters were set at 18 μm per layer and rotation step was set 0.7°. ROI for quantitative analysis was the weight-bearing area of the femoral head under the articular cortical bone. Three-dimensional (3D) image reconstruction was created and morphometric parameters were evaluated, including bone mineral density (BMD), bone volume to total volume fraction (BV/TV), bone surface/bone volume fraction (BS/BV), trabecular number (Tb.N), trabecular thickness (Tb.Th), and trabecular separation (Tb.Sp). To better assess the severity of femoral-head collapse, the area between the upper edge of the femoral head and the tangent circle is calculated in the coronal image. In addition, the cortical change of the femoral head was also recorded.

### Angiography

The anesthetized rats were fixed on the operating table. After exposing the abdominal aorta and arterial catheter indwelling, heparin saline and formalin was injected under appropriate pressure to complete vascular irrigation and fixation. Then, for each rat, 20 ml micro-fil (Flow Tech, Inc., Carver, MA, USA) was injected slowly at a uniform speed, and rats were sacrificed during this process. The femoral heads were removed after 12 h storage at 4° to make sure of complete polymerization of the contrast agent. Then, the decalcified femoral head was scanned and analyzed with the above-mentioned micro-CT, and a 3D model of femoral-head angiography was created.

### Histological, immunohistochemical, and immunofluorescence staining

Femoral-head samples went through fixation for 48 h in 10% formalin and decalcification for 4 weeks in 10% ethylenediaminetetraacetic acid (EDTA, Sigma-Aldrich). Then, the samples were embedded in paraffin and cut into 6 μm thick slices. H&E staining was performed to observe the general view of specimens and to evaluate the trabecular structure. For TRAP staining, paraffin sections were stained with TRAP staining solution (tartrate buffer containing naphthol AS-BI phosphate and pararosaniline chloride) at 37 °C for 1 h in darkness, and counterstained with Fast Green. Histomorphometry analysis including BV/TV, BA/TA, osteocyte number, and TRAP-positive osteoclasts were completed. Section images were acquired using an Axiovert 40C optical microscope (Zeiss, Germany). IHC staining was performed to define the expression of CB2, as well as osteogenesis, vascular, and pathway-related markers. In brief, sections were dewaxed and gradient hydrated to retrieve antigen. Then primary antibodies including CB2, β-catenin, and CD31 and corresponding secondary antibodies (all from Abcam Cambridge, UK) were incubated. The chromogenic reaction was induced by a DAB Kit (Beyotime, China). Immunofluorescence staining was also performed to clarify the expression level and localization of certain targets. Primary antibodies like Runx2, OCN, and VEGF (Abcam) and corresponding fluorescent secondary antibodies (Abcam) were used. The tissue sections were observed with a fluorescence microscope. IHC-staining-positive cells and areas were measured using Bioquant Osteo 2017 and counted by two independent observers.

### Double calcein labeling

Rats were double-labeled with calcein (10 mg/kg, Sigma-Aldrich) through i.p. injection at 10 and 3 days before euthanasia. Undecalcified femoral heads were fixed and dehydrated before sectioning. Images of calcein labeling were visualized by a fluorescence microscope, and parameters including inter-label width and bone MAR were measured.

### TUNEL assay

TdT-mediated dUTP nick-end labeling (TUNEL) assay was performed with one-step TUNEL apoptosis assay kit (Beyotime Institute of Biotechnology) according to the manufacturer’s instructions.

### Human tissue

The human femoral heads were obtained from total hip arthroplasties in the First Affiliated Hospital of Soochow University, three samples in each group. The femoral neck fracture samples were used as the control group, and the samples from patients diagnosed GC-induced ONFH were set as observation group.

### Cell culture, osteoblast differentiation, and proliferation assay

Rat-BMSCs were extracted from the tibia and femur of the Sprague-Dawley rats. For osteogenic differentiation, dexamethasone, vitamin C, and 10 mM β-glycerophosphate were contained. HUVECs were purchased from the Sciencell Corporation (Shanghai, China). Cell Counting Kit-8 (CCK-8; Beyotime) was used to evaluate cytotoxicity. In addition to gradient concentration or time of MP, the high-dose MP environment was set 100 μM, and JWH133 1 μM, AM630 1 μM, ICG-001 10 μM. Bevacizumab was purchased from Roche and was diluted to 0.5 μg/mL.

### Western blot analysis

Proteins in BMSCs were obtained by lysing cells in radio immunoprecipitation assay (RIPA; Beyotime). Bicinchoninic Acid Kit for protein (BCA kit; Sigma-Aldrich) was used to quantify protein concentration. After protein was separated and transmitted to a polyvinylidene fluoride membrane (Bio-Rad Laboratories), membrane was blocked (Beyotime) and incubated with primary antibodies overnight at 4 °C, including CB2 (1:500), Runx2 (1:500), OCN (1:500), Osterix (1:500), pSer9-GSK-3β (1:500), total GSK-3β (1:2000), β-catenin (1:2000), and Axin2 (1:1000, all obtained from Abcam). After incubation with corresponding secondary antibody, the protein bands were captured by enhanced chemiluminescence (ECL; Sigma-Aldrich). Relative gray level was measured for quantitative analysis using Image Lab 3.0.

### Alkaline phosphatase and Alizarin red staining

Alkaline phosphatase (ALP) staining was performed for culture in osteogenic medium for 14 days, BCIP/NBT (Beyotime) working solution was used to incubate. Alizarin red staining (ARS) was performed for culture in osteogenic medium for 21 days, using ARS staining solution (Sciencell). Pictures of well plates and images captured by microscope were preserved as quantitative basis.

### Cell immunofluorescence staining

After osteogenic induction and intervention, cells were fixed. Then, 0.2% Triton X-100 (Beyotime) was added for cell permeabilization. Next, cells were blocked with QuickBlock Blocking Buffer (Beyotime), incubated with primary antibody of β-catenin and secondary antibody, and then counterstained with DAPI for 10 min. The results were observed with a fluorescence microscope, and fluorescence intensity was assessed using ImageJ.

### Transwell migration assay

HUVECs were preconditioned and were plated into the upper chambers of a transwell plate (Corning). Complete culture medium was used as a chemoattractant, and placed in the lower chamber. Then, 24 h later, the membranes were fixed and then stained with crystal violet (Beyotime), and the membranes were mounted and observed with light microscope.

### Scratch assay

HUVECs were cultured in 6-well plates to a confluent monolayer. Two separate wounds were scratched using a pipet tip and cells were rinsed with serum-free medium. Pictures at the same position of the wound were taken by microscope at 0, 12, and 24 h. Migration ability was analyzed by quantifying the wound-healing area using Image Pro Plus software (IPP, Media Cybernetics, Rockville, MD, USA).

### Tube formation

Tube formation was performed to evaluate capillary-like structure formation of HUVECs. HUVECs were washed twice with serum-free medium, then cells were suspended and intervened in serum-free medium, and plated on angiogenesis plates. The extent of tube formation was assessed 6 h after seeding. IPP software was used to quantify tube length and branch points.

### Statistical analysis

All experiments were repeated twice with at least three replications. The results were expressed as mean ± standard deviation (SD), GraphPad Prism 7.0 (GraphPad Software, Inc., USA) was used to analyze. One-way ANOVA is used to determine statistical significance. Differences were considered significant with *p* < 0.05.

## Supplementary information

Supplemental material

## Data Availability

The datasets used and/or analyzed during the current study are available from the corresponding authors on reasonable request.
