# Supplementary material for: Activation of cannabinoid receptor 2 alleviates glucocorticoid-induced osteonecrosis of femoral head with osteogenesis and maintenance of blood supply
Source: Cell Death Dis. 2021 Oct 30;12(11):1035. doi: 10.1038/s41419-021-04313-3 (PMC8556843; doi:10.1038/s41419-021-04313-3)
Supplement: Supplementary file 1 — Supplemental material [file 41419_2021_4313_MOESM1_ESM.docx]

**Supplemental Material**


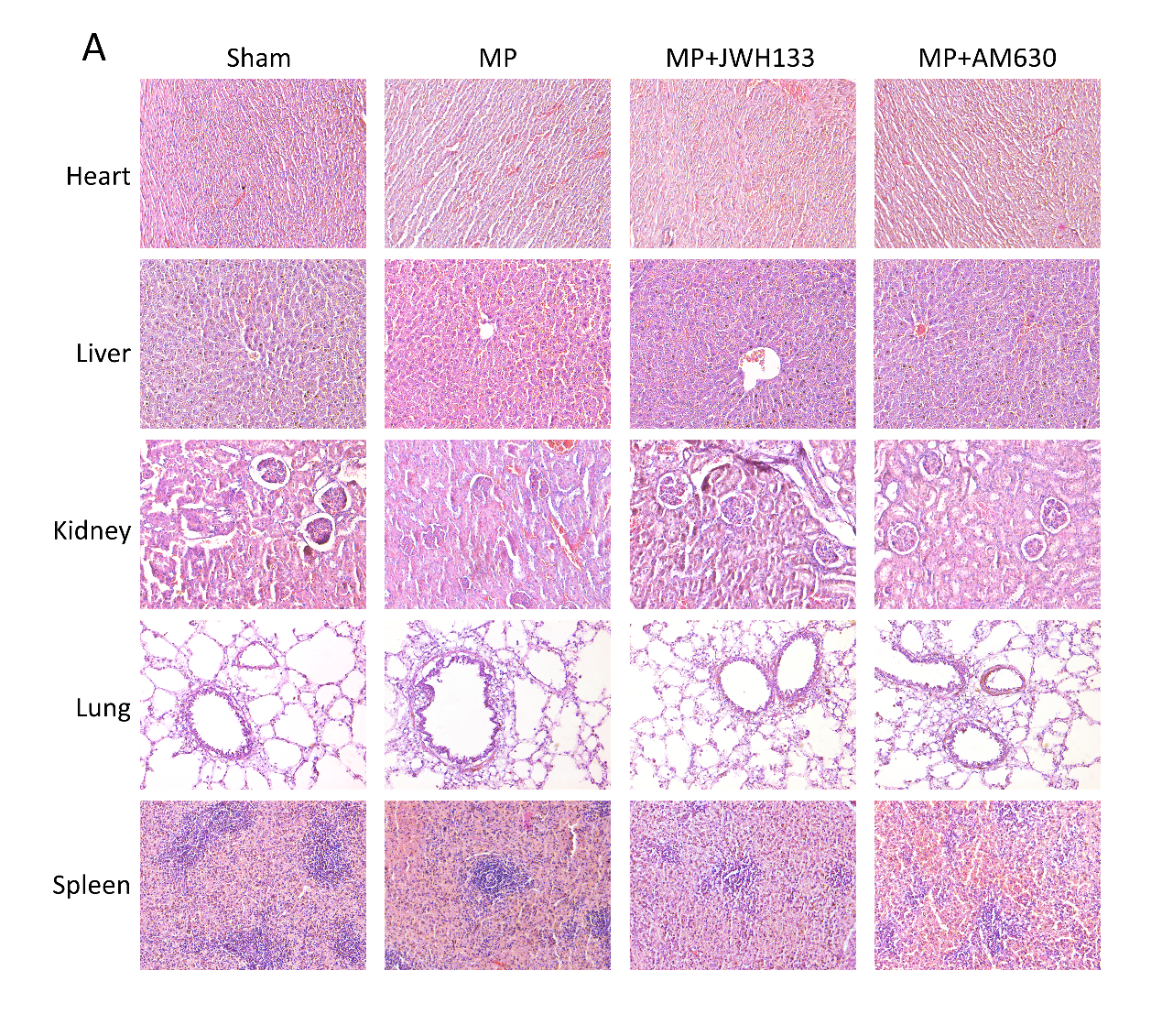


**Figure. Sup1.** (A) Toxicities evaluation on heart, liver, spleen, kidney, lung and spleen. H&E staining of the organ tissue sections.


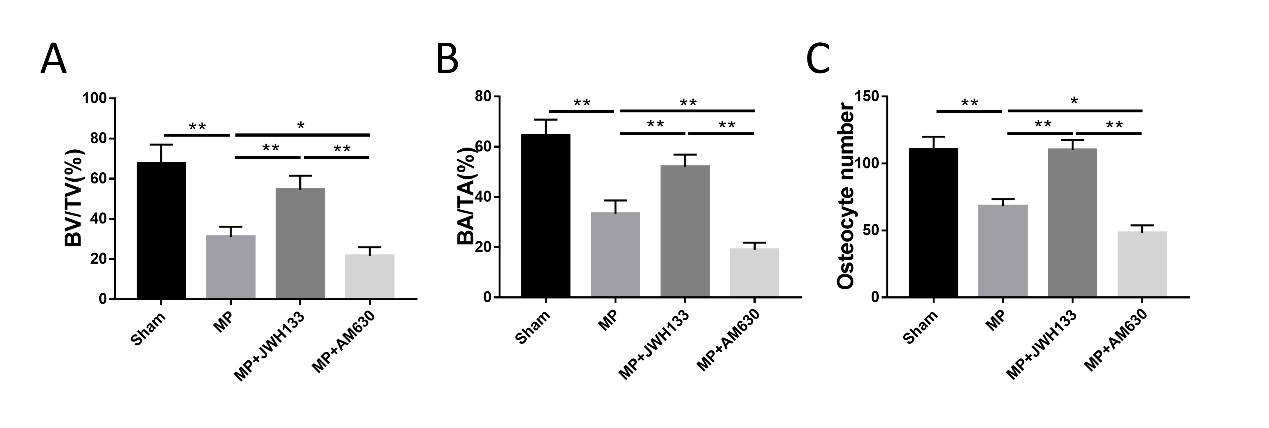


**Figure. Sup2.** (A) Histomorphometric analyses of bone parameters BV/TV (%), (B) Histomorphometric analyses of bone parameters BA/TA (%), (C) Quantitative analyses of osteocyte number. (n=10 per group. Data are showed as means ± SD, *p<0.05 and **p<0.01)


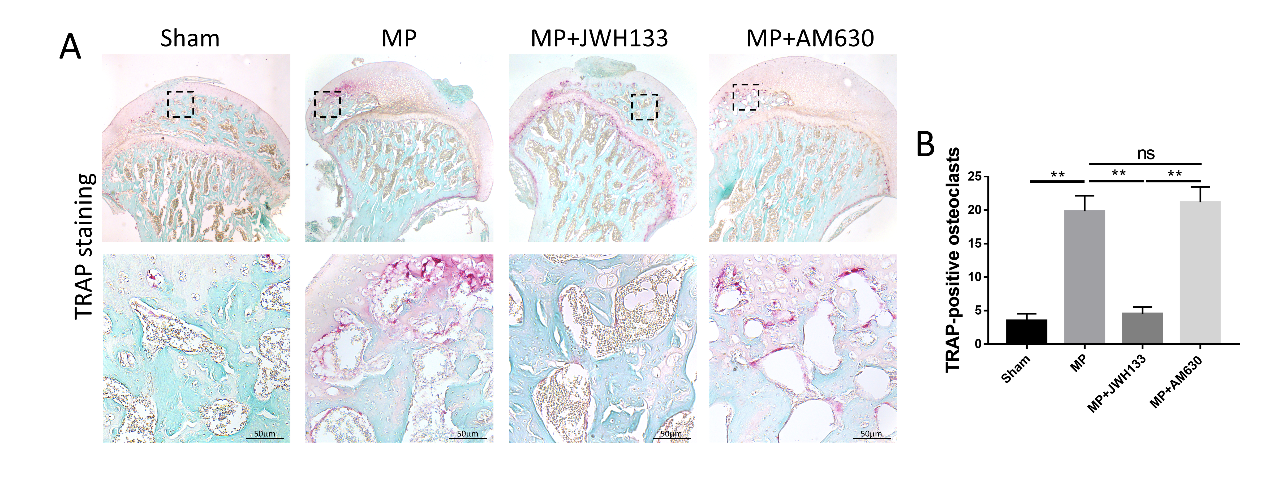


**Figure. Sup3.** (A) TRAP staining of femoral head, (B) Quantitative analyses of osteoclast number. (n=10 per group. Data are showed as means ± SD, *p<0.05; **p<0.01; *ns*, not significant)


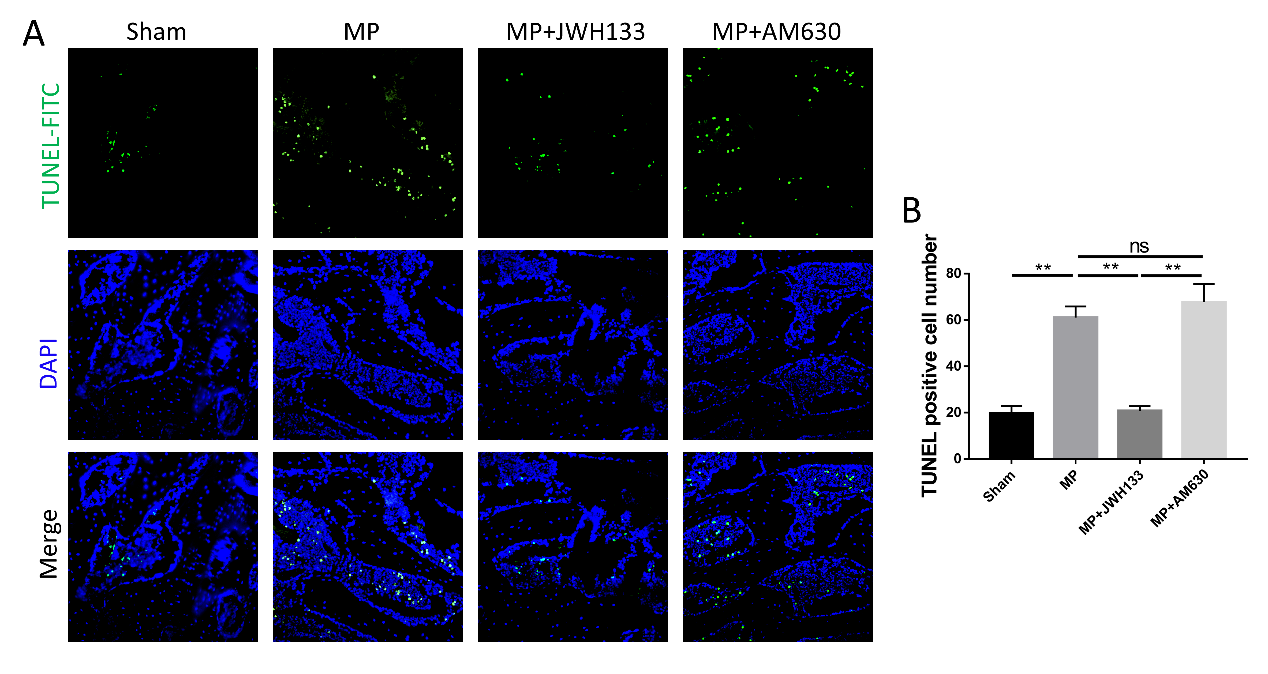


**Figure. Sup4.** (A) TUNEL staining of femoral head. (B) Quantitative analyses of apoptotic osteocytes. (n=10 per group. Data are showed as means ± SD, *p<0.05; **p<0.01; *ns*, not significant)


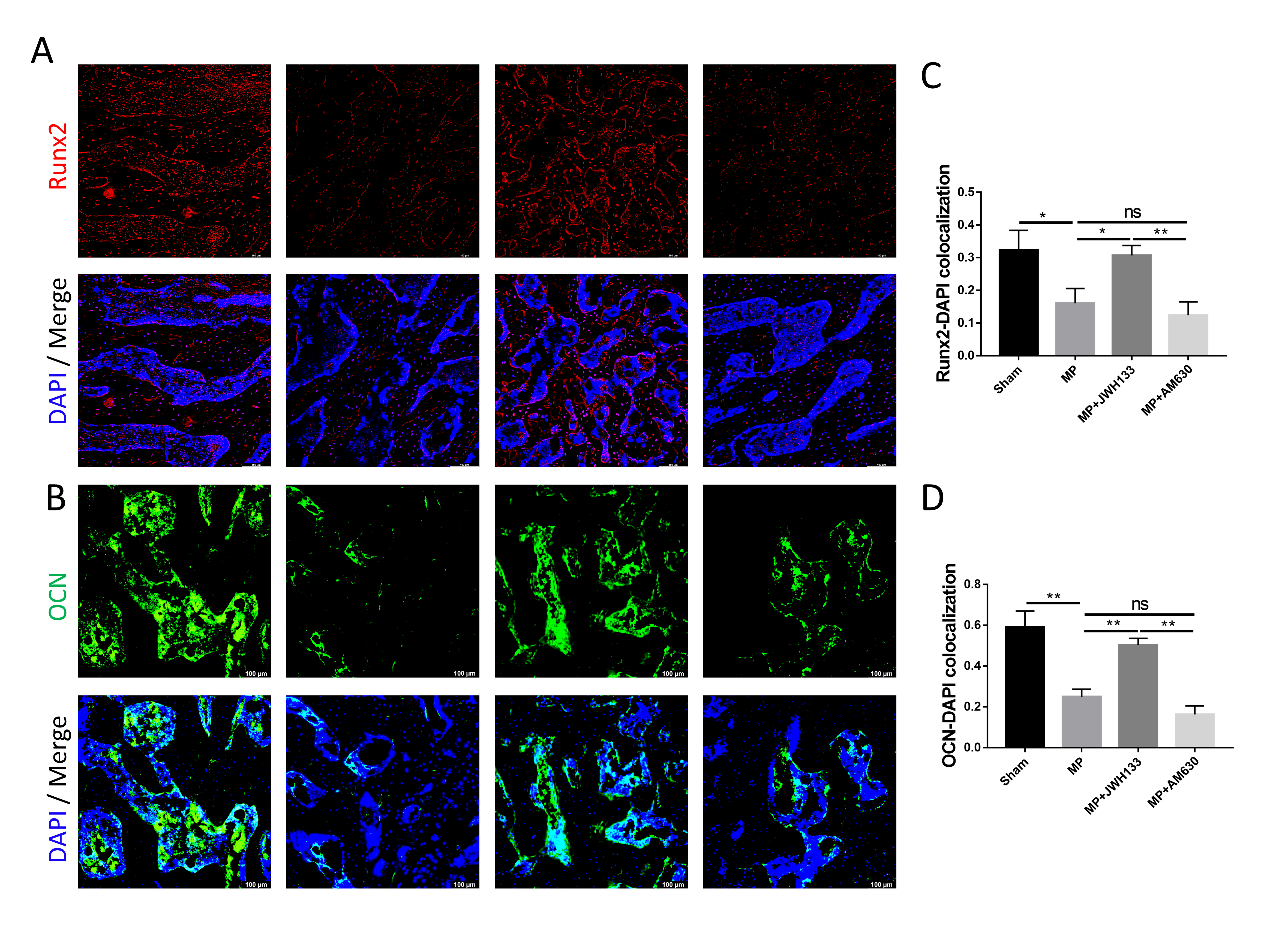


**Figure. Sup5.** (A, B) Immunofluorescence and quantitative analyses of Runx2, (C, D) Immunofluorescence and quantitative analyses of OCN. (n=10 per group. Data are showed as means ± SD, *p<0.05; **p<0.01; *ns*, not significant)


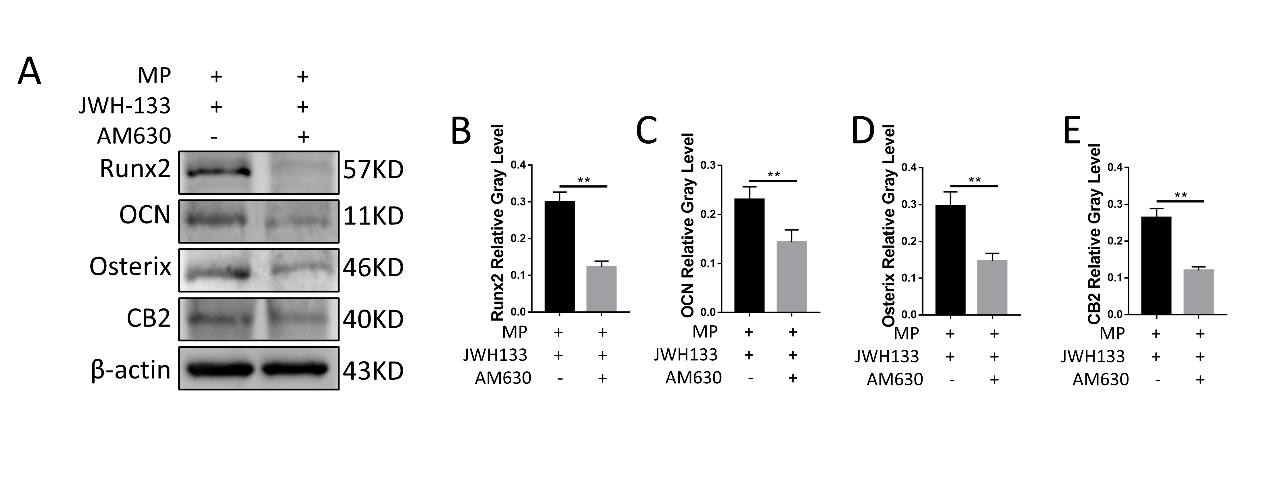


**Figure. Sup6.** (A) Protein expression levels of Runx2, OCN, Osterix, and CB2, (B-E) quantitative analysis of Runx2, OCN, Osterix, and CB2 expression. (n=3 per group. Data are showed as means ± SD. *p<0.05; **p<0.01; *ns*, not significant)


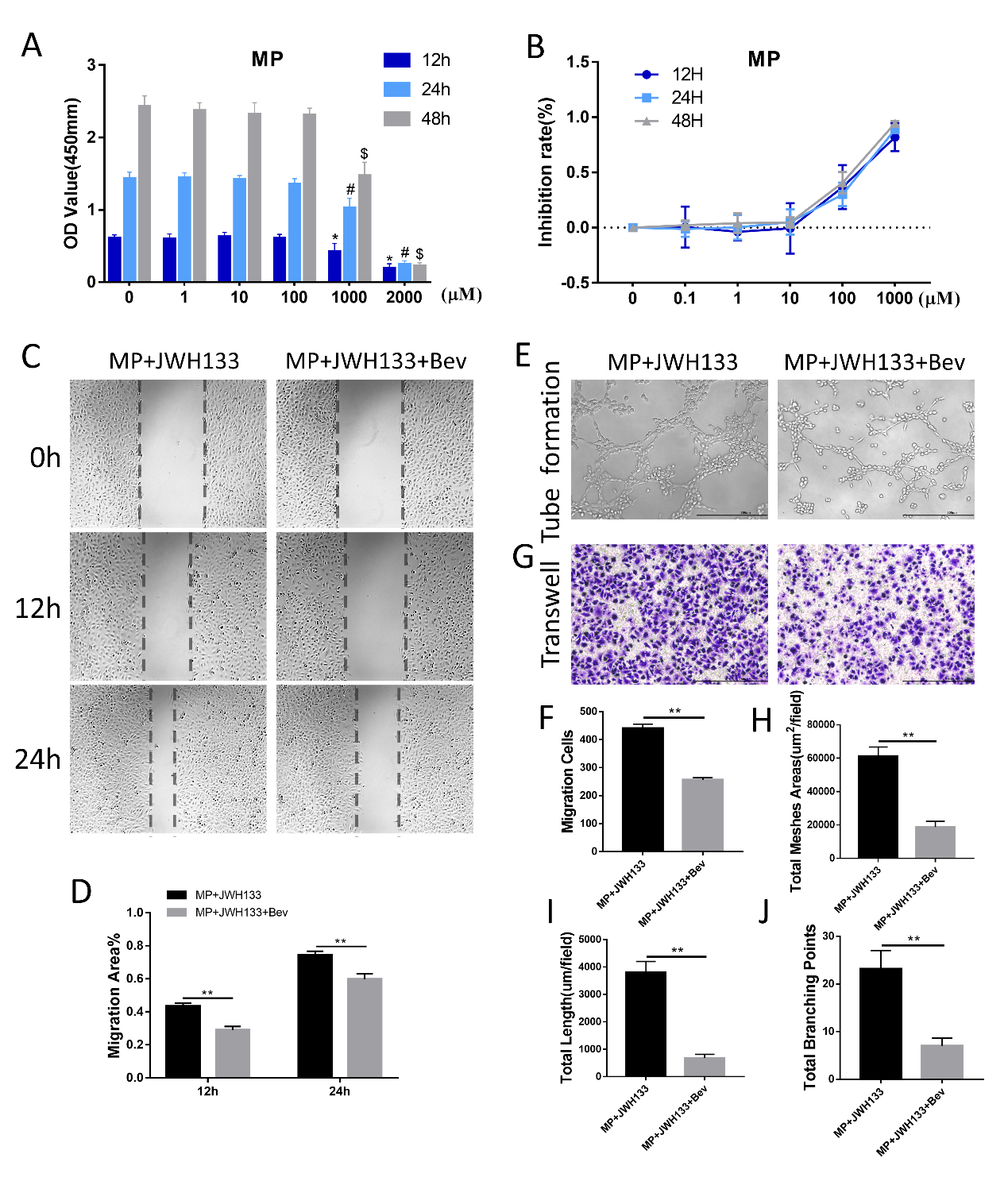


**Fig. Sup7.** Bevacizumab blunted the vasogenic effect of JWH133 in vitro. (A, B) CCK-8 assay of MP in HUVECs. *p<0.05, compared with control group (12h), #p<0.05, compared with control group (24h), and $p<0.05, compared with control group (48h). (C, D) Scratch assay for 0h, 12h, and 24h. (E, F) Transwell assay, and migration cells. (G-J) Tube formation and quantitative analysis. (n=3 per group. Data are showed as means ± SD, *p<0.05; **p<0.01; *ns*, not significant)
